# Frontonasal Encephalocele Complicated With Pseudotumor Cerebri: A Case Report and Literature Review

**DOI:** 10.7759/cureus.45509

**Published:** 2023-09-18

**Authors:** Alaa Hamad, Dalal F Alageel, Abdul Rahman Khan, Faisal Joueidi, Anas S Alyazidi, Atiah B Ismail, Peter Spangenberg, Imaduddin Kanaan

**Affiliations:** 1 College of Medicine, Alfaisal University, Riyadh, SAU; 2 College of Medicine, King Abdulaziz University, Riyadh, SAU; 3 Neurosurgery, King Faisal Specialist Hospital and Research Centre, Riyadh, SAU

**Keywords:** literature review, case report, encephalocele, frontal skull base reconstruction, idiopathic intracranial hypertension, frontonasal encephalocele, pseudotumor cerebri syndrome

## Abstract

Primary pseudotumor cerebri syndrome (PPTS) is a rare disorder of elevated intracranial pressure (ICP) in the absence of an identifiable underlying etiology. Afflicted patients are usually obese women in their reproductive age presenting with symptoms of elevated ICP. Seldom, patients can present with an encephalocele. We reported a case of a 31-year-old female who initially presented to our center with complaints of headaches, foreign body sensation in the nasal cavity, and decreased ability to smell. Brain computed tomography (CT) scan showed a large intranasal encephalocele and defect along the frontal skull base, through which brain tissue was herniating. The patient was successfully treated surgically by implantation of a lumboperitoneal shunt to manage the high ICP caused by her PPTS. In combination, reconstruction of the frontal skull base defect for the encephalocele was performed. Currently, the patient is doing well despite some on-and-off headaches.

## Introduction

Primary pseudotumor cerebri syndrome (PPTS), alternatively known as idiopathic intracranial hypertension is a rare disorder of elevated intracranial pressure (ICP) in the absence of an identifiable underlying etiology [1]. PPTS is relatively rare with an estimated incidence rate of 19 per 100,000 cases among obese patients aged 20-40 years [2]. Patients can be diagnosed with PPTS following radiological exclusion of alternative underlying causes of ICP, such as mass lesions, malignancies, or infections. Despite several theories and efforts to elucidate the pathophysiology of PPTS, the precise pathogenesis remains unknown. However, published studies presented and established a strong association between PPTS and obese women of reproductive age. Such patients usually present clinically with symptoms of elevated ICP. Classically, presenting symptoms include headaches, visual changes, and back pain [3]. Seldom, patients can present with cerebrospinal fluid (CSF) rhinorrhea, or in extremely rare cases, encephaloceles [3,4].

Encephaloceles, conditions usually arise because of the chronic applied pressure between areas of arachnoid granulations and venous sinuses with the skull base. The constant pressure consequently leads to the weakening of bones and results in the dehiscence of the skull base and the formation of an aperture between the cranium and the sinonasal tract. This results in herniation of the brain tissue and the formation of an encephalocele [4]. The diagnosis is by exclusion and using the modified Dandy criteria. The incidence rate has been increasing significantly in the past decades. Thus, the annual incidence rate in the USA is estimated to be 0.9-1/100,000 in the general population [1]. Encephaloceles are generally classified according to the location of the skull defect through which they herniate. Among different locations of the skull defects, a frontonasal encephalocele is a result of a frontoethmoidal skull defect through which the brain tissue herniates into the nasal cavity [4]. Despite recent literature emphasizing a decline in the use of surgical intervention and prompting medical treatment [5], in this article, we report a case of a frontonasal encephalocele in a young 31-year-old female diagnosed with PPTS and successfully treated surgically with the implantation of a lumboperitoneal shunt in combination with the reconstruction of the frontal skull base defect.

## Case presentation

**Figure 1:**
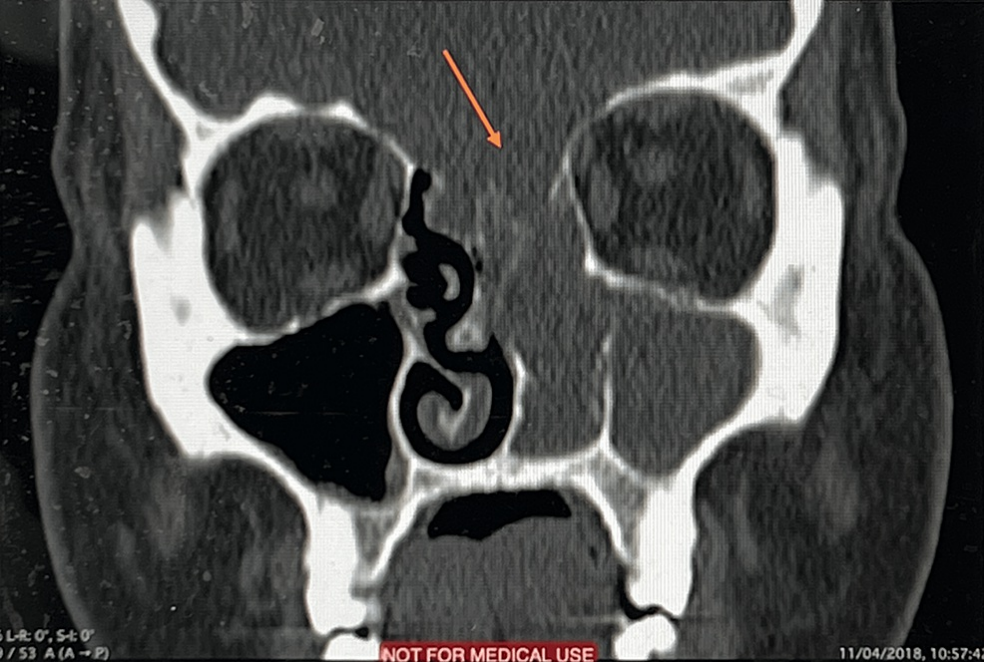
Coronal computed tomography showing skull base defect.

Following CARE guidelines for reporting case reports [6], we report a case of a 31-year-old obese female patient with a body mass index (BMI) of 37.2 who initially presented to our center with a nine-month history of foreign body sensation in the nasal cavity, progressive nasal obstruction, occasional watery nasal discharge, hyposmia, and persistent frontal headache. The patient had no history of head trauma or surgeries. A nasopharyngeal examination revealed a mass emerging from the left nasal cavity with complete obstruction. Coronal section computed tomography (CT) revealed a skull defect through which an encephalocele was protruding through the frontoethmoid (Figure 1). Following the aforementioned radiological finding, the patient was referred to neurosurgery.

Two weeks later, and while waiting for her neurosurgery appointment, she presented to the emergency department with a three-day history of fever, headache, photophobia, nuchal rigidity, severe neck pain, and vomiting. Physical examination findings and laboratory results were unremarkable. A head CT scan was conducted and a new area of hypo-density was visualized concerning cerebritis. The patient was referred to the hospital’s infectious diseases (ID) team where 2g IV of empiric ceftriaxone for 14 days and a one-time 1000 mg of IV vancomycin therapy was initiated. An urgent lumbar puncture was also highly recommended by the ID team. Results of the lumbar puncture demonstrated an opening pressure of 48 cm H_2_O in the horizontal position, and surprisingly, the CSF analysis results revealed clear CSF, high protein, normal glucose, and a negative bacterial culture; therefore ruling out meningitis. The clinical presentation of a high BMI in a young female and high ICP without an identifiable underlying causative etiology raised clinical suspicion for pseudotumor cerebri. Therefore, a magnetic resonance image (MRI) was performed and showed mildly dilated CSF spaces around the optic nerves and a bulging CSF space into the sella, confirming pseudotumor cerebri. The patient was finally diagnosed as a case of PTTS with a frontonasal bone defect resulting in brain herniation into the nasal cavity and nasopharynx.

**Figure 2:**
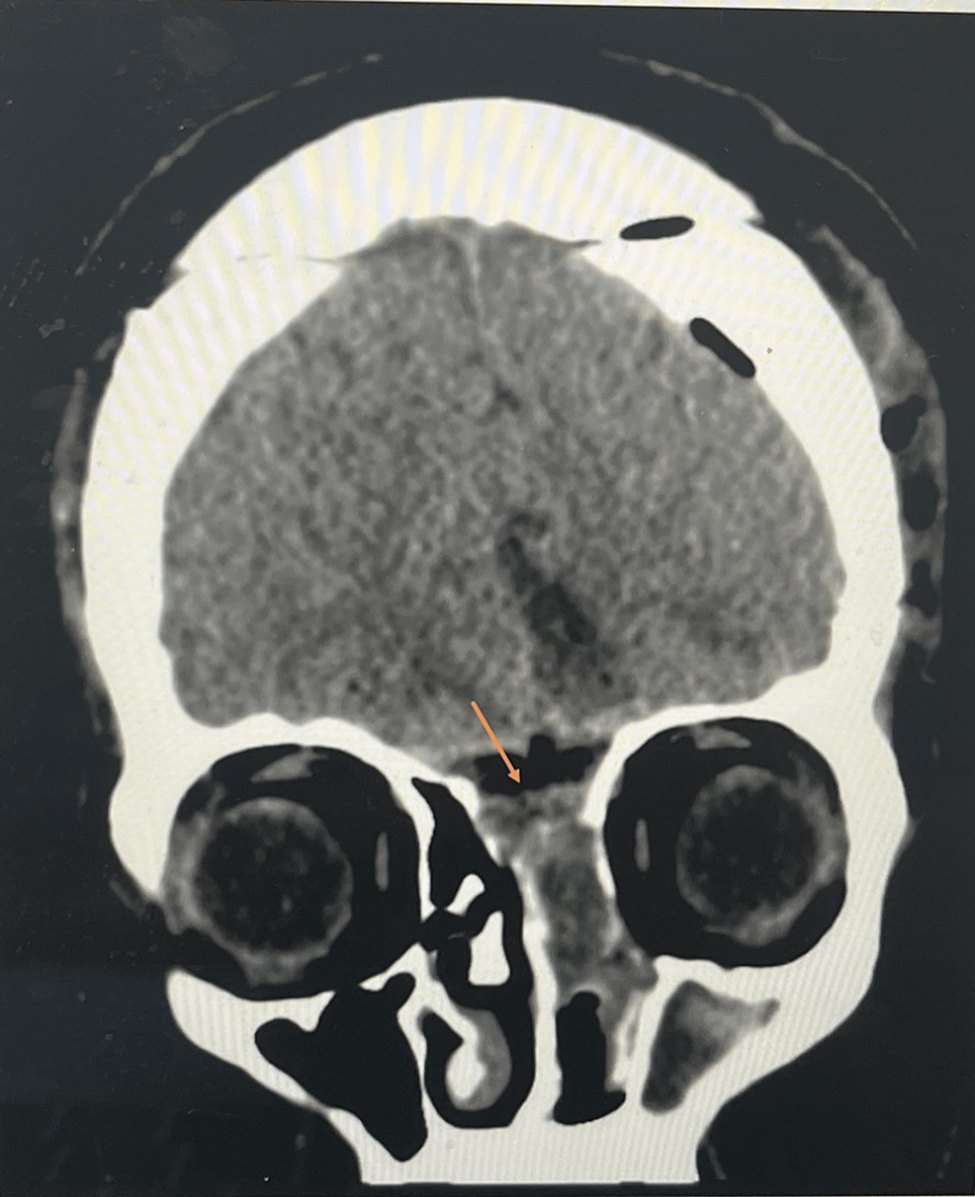
Coronal brain computed tomography without contrast.

The size of the encephalocele was too large and symptomatic to manage conservatively. Therefore, the patient was subsequently scheduled for surgical repair of the encephalocele and management of the high ICP. The patient initially underwent a bifrontal craniotomy, where the frontal skull base was examined through a microscope that allowed extradural identification of the skull defect. The dura was then opened, and the herniating brain tissue was coagulated and transected. The dural compartment was finally closed with duraplasty, and the bone defect closed with bone cement yielding a very strong reconstructed skull base. The patient was then re-prepped, and the herniating tissue was removed gradually trans-nasally as seen in the coronal section CT scan (Figure 2). The lumbar peritoneal shunt was also inserted to relieve the high ICP.

Postoperatively the patient is doing well with occasional episodes of headache but had no nausea, vomiting, or visual loss. The patient, however, continued to suffer from anosmia which improved to hyposmia in a month’s duration. In an attempt to lower the patient’s high BMI, the patient is planning to undergo gastric sleeve surgery for weight loss. The patient is currently following up with neurosurgery where her symptoms and skull base are being assessed on an annual basis through MRI.

## Discussion

Pseudotumor cerebri is a condition characterized by elevated ICP [3]. Despite the fact that an exact cause has not been established, PTTS has been strongly associated with risk factors such as obesity and the female gender as observed in our patient [7]. The most classical and typical symptom of PTTS is headache, occurring in over 90% of patients. This symptom was found in our patient, which, along with the clinical presentation and radiologic findings, supported our suspicion of the disease. PTTS has also been shown to be associated with encephaloceles [8]. Several hypotheses surround this association, however, most notably one that hypothesizes that encephaloceles develop because of prolonged pressure that continuously exerts pressure and weakens the bones. Eventually, this causes a gap to form between the cranium and the sinonasal tract and consequent herniation of the brain [2]. To the extent of our knowledge, there have only been a few documented cases of an encephalocele secondary to PPTS [9-11]. Table 1 compares and contrasts the different cases in the literature to this case.

**Table 1:** Descriptive summary of cases We summarize the cases with demographics, presenting symptoms, laboratory investigations, radiological findings, surgical procedures, and outcomes.

Birth details and dysmorphic features	Present case	Zhang et al. [9]	Lau and Hassan [10]	Thottiyil et al. [11]
Reported country	Saudi Arabia	China	Malaysia	India
Year of reporting	2023	2022	2022	2022
Sex	Female	Female	Female	Female
Age	31 years	54 years	44 years	26 years
BMI	37.2	NM	NM	23
Initial complaint	Left nasal obstruction	Fever, nausea and vomiting	Headache, eye pain with blurred vision	Seizure
Constitutional symptoms				
Fever	+	+	NM	-
Nausea	-	+	NM	-
Vomiting	-	+	NM	-
Nervous system				
Headache	+	+	+	+
Photophobia	+	-	-	-
Nuchal rigidity	+	+	-	-
Nasopharyngeal				
Nasal discharge	+	+	NM	-
Hyposmia	+	+	NM	-
Radiological				
Abnormal imaging	+	+	+	+
Laboratory				
WBC	High	Normal	NM	-
CSF	Normal	Normal	-	+
Surgery				
Underwent surgical repair	+	-	NM	ND
Post-surgical outcome	Positive	N/A	NM	ND
NM: Not mentioned; N/A: not available; WBC: whole blood count; CSF: cerebrospinal fluid				

Zhang et al. (2022) reported a case of a 54-year-old Chinese woman with PTTS and concomitant age-related bone degeneration. They believe that the patient was suffering from PTTS for many years, but the diagnosis was missed due to an atypical presentation lacking the characteristic CSF [9]. They discuss chronic elevated ICP as a cause of an enlargement of a preexisting skull defect. As a consequence of this enlargement, an aperture developed leading to the encephalocele. In contrast, our patient, a young 31-year-old female with a symptom onset of a period of nine months presented initially as nasal obstruction. The patient later had a CT done and was diagnosed with an encephalocele. This case was additionally one of the very few where a patient is managed surgically. Nevertheless, due to the similarity of these two cases, we strongly believe that the association between chronic pressure in the brain and encephaloceles should be investigated further.

## Conclusions

In this case, we shed light on the importance of suspicion of PTTS in the case of recurrent episodes of rhinorrhea in obese women of childbearing age and untraceable causes of high ICP. We believe that possible complications such as encephalocele could be potentially avoided if provided early intervention. We encourage future cases of PTTS to be reported, as it is a rare disorder, and our atypical case may be more common than what is reported in the literature. In the context of our case and what has been reported previously, we believe that PTTS is a potential cause of encephalocele however to establish an association more research needs to be conducted.
